# Alpha-interferon does not increase the efficacy of 5-fluorouracil in advanced colorectal cancer

**DOI:** 10.1054/bjoc.2000.1669

**Published:** 2001-03

**Authors:** Meta-Analysis Group in Cancer

**Keywords:** 5-fluorouracil, interferon, colorectal cancer, meta-analysis

## Abstract

Two meta-analyses were conducted to quantify the benefit of combining α-IFN to 5FU in advanced colorectal cancer in terms of tumour response and survival. Analyses were based on a total of 3254 individual patient data provided by principal investigators of each trial. The meta-analysis of 5FU ± LV vs. 5FU ± LV + α-IFN combined 12 trials and 1766 patients. The meta-analysis failed to show any statistically significant difference between the two treatment groups in terms of tumour response or survival. Overall tumour response rates were 25% for patients receiving no α-IFN vs. 24% for patients receiving α-IFN (relative risk, RR = 1.02), and median survivals were 11.4 months for patients receiving no α-IFN vs. 11.5 months for patients receiving α-IFN (hazard ratio, HR = 0.95). The meta-analysis of 5FU + LV vs. 5FU + α-IFN combined 7 trials, and 1488 patients. This meta-analysis showed an advantage for 5FU + LV over 5FU + α-IFN which was statistically significant in terms of tumour response (23% vs. 18%; RR = 1.26;*P* = 0.042), and of a borderline significance for overall survival (HR = 1.11;*P* = 0.066). Metastases confined to the liver and primary rectal tumours were independent favourable prognostic factors for tumour response, whereas good performance status, metastases confined to the liver or confined to the lung, and primary tumour in the rectum were independent favourable prognostic factors for survival. We conclude that α-IFN does not increase the efficacy of 5FU or of 5FU + LV, and that 5FU + α-IFN is significantly inferior to 5FU + LV, for patients with advanced colorectal cancer. © 2001 Cancer Research Campaign http://www.bjcancer.com


*British Journal of Cancer* (2001) **84**(5), 611–620
© 2001 Cancer Research Campaign
doi: 10.1054/ bjoc.2000.1669, available online at http://www.idealibrary.com on IDEAL

http://www.bjcancer.com

### Alpha-interferon does not increase the efficacy of 5-fluorouracil in advanced colorectal cancer

**Meta-Analysis Group in Cancer**

**Summary** Two meta-analyses were conducted to quantify the benefit of combining α-IFN to 5FU in advanced colorectal cancer in terms of tumour response and survival. Analyses were based on a total of 3254 individual patient data provided by principal investigators of each trial. The meta-analysis of 5FU ± LV vs. 5FU ± LV + α-IFN combined 12 trials and 1766 patients. The meta-analysis failed to show any statistically significant difference between the two treatment groups in terms of tumour response or survival. Overall tumour response rates were 25% for patients receiving no α-IFN vs. 24% for patients receiving α-IFN (relative risk, RR = 1.02), and median survivals were 11.4 months for patients receiving no α-IFN vs. 11.5 months for patients receiving α-IFN (hazard ratio, HR = 0.95). The meta-analysis of 5FU + LV vs. 5FU + α-IFN combined 7 trials, and 1488 patients. This meta-analysis showed an advantage for 5FU + LV over 5FU + α-IFN which was statistically significant in terms of tumour response (23% vs. 18%; RR = 1.26; $P = 0.042$), and of a borderline significance for overall survival (HR = 1.11; $P = 0.066$). Metastases confined to the liver and primary rectal tumours were independent favourable prognostic factors for tumour response, whereas good performance status, metastases confined to the liver or confined to the lung, and primary tumour in the rectum were independent favourable prognostic factors for survival. We conclude that α-IFN does not increase the efficacy of 5FU or of 5FU + LV, and that 5FU + α-IFN is significantly inferior to 5FU + LV, for patients with advanced colorectal cancer. © 2001 Cancer Research Campaign http://www.bjcancer.com

**Keywords**: 5-fluorouracil; interferon; colorectal cancer; meta-analysis

The outcome of patients with non-operable metastatic colorectal cancer remains poor. Four meta-analyses previously performed by the *Meta-Analysis Group In Cancer* confirmed that the effect of intravenous bolus 5-fluorouracil (5FU) can be increased by the modulation of 5FU by leucovorin (Advanced Colorectal Cancer Meta-analysis Project 1992) or by methotrexate (Advanced Colorectal Cancer Meta-analysis Project 1994), the administration of 5FU by continuous infusion (Meta-Analysis Group in Cancer 1998), or the administration of fluoropyrimidines through the hepatic artery (Meta-Analysis Group in Cancer 1996) in case of metastases confined to the liver. Each meta-analysis showed a large increase in tumour response, without substantial impact on survival.

In the late 1980s, alpha-interferon (α-IFN) was proposed to increase the efficacy of 5FU in advanced colorectal cancer. After the initial report by Wadler et al (1989) of a tumour response rate of 76% in a group of 17 previously untreated patients, additional phase II trials of 5FU plus α-IFN with or without leucovorin were undertaken (Pazdur et al, 1990); (Piedbois et al, 1991); (Weh et al, 1992); (Raderer and Scheithauer, 1995) followed by several randomized phase III trials. Most randomized trials were disappointing, but despite a total of 3500 patients enrolled in these studies, there is to date no overall assessment of the true impact of α-IFN in advanced colorectal cancer. We therefore decided to explore this question through a meta-analytic approach based on individual patient data. Toxicity was not studied, since at the time of beginning the present analyses, individual trials had already demonstrated that the addition of α-IFN to a 5FU regimen led to an increased risk of toxicity.

*Received 12 July 2000*
*Revised 30 October 2000*
*Accepted 21 November 2000*

*Correspondence to:* Pascal Piedbois, MD, Department of Medical Oncology, Henri Mondor Hospital, Assistance Publique Hôpitaux de Paris, 94000 Creteil, France (Tel. 33 1 49 81 25 82; Fax. 33 1 49 81 25 69; E-mail:pascal.piedbois@hmn.ap-hop-paris.fr)

**Writing Committee**:
Pierre Thirion, Pascal Piedbois, Marc Buyse, Peter J. O'Dwyer, David Cunningham, Anthony Man, Frank A. Greco, Giuseppe Colucci, Claus-Henning Köhne, Francesco Di Costanzo, Andrea Piga, Sergio Palmeri, Patrick Dufour, Allessandra Cassano, Gabor Pajkos, Raul Pensel, N. Faruk Aykan, John Marsh, Matthew T. Seymour

**Collaborators**: Peter J. O'Dwyer, Louise Ryan, Judith Manola (Eastern Cooperative Oncology Group, Cancer and Leukemia Group B, USA), David Cunningham, Andy Norman (The Royal Marsden Hospital, Sutton, United Kingdom), Matthew T. Seymour, Richard J. Stephens, (Medical Research Council, United Kingdom), Giuseppe Colucci (Gruppo Oncologico dell' Italia Meridionale, Italy), Claus-Henning Köhne, Hans-Joachim Schmoll, (Arbeitsgemeinschaft Internische Onkologie, Germany), Francesco Di Costanzo (Gruppo Oncologico Italiano di Ricerca Clinica, Italy), Andrea Piga (University of Ancona, Italy), Sergio Palmeri, (University of Palermo, Italy), Patrick Dufour (Hôpital de Hautepierre, Strasbourg, France), Allessandra Cassano, Carlo Barone, (Universita Catolica S. Cuore, Roma, Italy), Anthony Man (Novartis Pharma, Basel, Switzerland), Frank A. Greco (Sarah Cannon Cancer Center, Nashville, TN, USA), Lawrence Einhorn, (Indiana University Cancer Center, Indianapolis, USA), Gabor Pajkos (MI Central Hospital, Budapest, Hungary), Gyorgy Bodoky (National Institute of Oncology, Budapest, Hungry), Raul Pensel (Hospital Municipal Jose M. Penna, Buenos Aires, Argentina), N. Faruk Aykan, (Istanbul University, Turkey, John Marsh (Yale University School of Medicine, New Haven, CT, USA), Peter Sorensen (Aarhus University Hospital, Aarhus, Denmark), Paris Kosmidis (Helenic Cooperative Oncology Group, Greece), Francesco Recchia (Istituto Oncologico Regione Abruzzo e Molise, Italy), Pierre Thirion (St. Luke's Hospital, Dublin, Ireland), Youri Piedbois, Eric Gauthier, Anne-Chantal Braud, Alain Piolot (European Association for Research In Oncology, Creteil, France), Pascal Piedbois (Henri Mondor Hospital, Assistance Publique de Paris, Creteil, France), Marc Buyse, Emmanuel Quinaux (International Drug Development Institute, Brussels, Belgium)

***Board of the Meta-Analysis Group In Cancer:*** Norman Wolmark (Pittsburgh, PA, USA) (President), Pascal Piedbois, MD (Creteil, France) (Secretary), Marc Buyse, ScD (Brussels, Belgium) (Statistician), Charles Erlichman, MD (Rochester, MN, USA), Robert Carlson, MD (Stanford, CA, USA), Youssef Rustum, PhD (Buffalo, NY, USA).

### METHODS

### Trial selection

Two meta-analyses were conducted concomitantly. In the first one we considered all properly randomised trials comparing 5FU with or without folinic acid (5FU ± LV) to the same 5FU ± LV regimen plus α-IFN (5FU ± LV + α-IFN). In the second meta-analysis we considered all properly randomised trials comparing 5FU + LV to 5FU + α-IFN. In both meta-analyses, α-IFN must have consisted of α-2a-interferon or α-2b-interferon, and patients must have been included in the trial before July 1996. The search for relevant trials was initiated in October 1996 by consulting MEDLINE, Physician Data Query (PDQ), the proceedings of major conferences since 1989, and through contacts with principal investigators. A total of 20 relevant trials were identified, but 3 of them (335 patients) could not be included in the meta-analysis, due to lack of data or information on the trial (Kreuser et al, 1995); (Kosmidis et al, 1996); (Recchia et al, 1996).

### Meta-analysis of 5FU ± LV vs. 5FU ± LV + α-IFN (Table 1)

The comparison of 5FU versus 5FU + α-IFN was addressed in 7 trials, the Roche International Clinical Research Center (RICRC) trial (Greco et al, 1996), the Palermo trial (Palmeri et al, 1998), the Ancona trial (Piga et al, 1996), two Royal Marsden Hospital (RMH) trials (Hill et al, 1995a+b), the trial from France (Dufour et al, 1996), and the Eastern Cooperative Oncology Group, Cancer and Leukemia Group B (ECOG/CALGB) trial (O'Dwyer et al, 1996). The ECOG/CALGB trial (O'Dwyer et al, 1996) was not considered in the first meta-analysis, because unlike the other trials, the planned dose of 5FU and its mode of administration were not the same in the 2 treatment groups. In most trials, the 5FU regimen was close to the Wadler regimen (Wadler et al, 1989), consisting of an initial 5-day 5FU infusion followed by a weekly 5FU infusion. The dose of 5FU varied from 500 to 750 mg/m$^2$/day. The dose of α-IFN varied from 3 to 10 MU, 3 times a week. Based on the impact of the mode of 5FU administration on tumour response and survival (Meta-Analysis Group In Cancer,

**Table 1** Randomised clinical trials comparing 5FU ± LV to 5FU ± LV + α-IFN in advanced colorectal cancer

| Comparison | Patients | Treatment arms |
|---|---|---|
| **5FU vs. 5FU + α-IFN, with 5FU bolus** | | |
| RICRC<br>Greco et al, 1996 | 245 | 5FU 750 mg/m$^2$/d continuous infusion d1 to d5, then weekly on bolus<br>Same + α-IFN 9 MU three times a week |
| Palermo<br>Palmeri et al, 1998 | 169 | 5FU 750 mg/m$^2$/d bolus d1 to d5; then weekly<br>Same + α-IFN 9 MU three times a week |
| Ancona<br>Piga et al, 1996 | 141 | 5FU 500 mg/m$^2$/d bolus d1 to d5; then weekly<br>Same + α-IFN 3 MU/d |
| RMH<br>Hill et al, 1995a | 106 | 5FU 750 mg/m$^2$/d continuous infusion d1 to d5; then weekly on bolus<br>Same + α-IFN 10 MU three times a week |
| France<br>Dufour et al, 1996 | 106 | 5FU 750 mg/m$^2$/d continuous infusion d1 to d5; then weekly on bolus<br>Same + α-IFN 9 MU three times a week |
| **Comparison** | **Patients** | **Treatment arms** |
| **5FU vs. 5FU + α-IFN, with 5FU continuous infusion** | | |
| RMH PVI<br>Hill et al, 1995b | 160 | 5FU 300 mg/m$^2$/d continuous infusion d1 to d70 followed by a 2 week-break<br>Same + α-IFN 5 MU three times a week |
| **Comparison** | **Patients** | **Treatment arms** |
| **5FU + LV vs. 5FU + LV + α-IFN, with 5FU bolus** | | |
| GOIM<br>Colucci et al, 1999 | 204 | 5FU 375 mg/m$^2$/d bolus d1 to d5, + l-folinic acid 100 mg/m$^2$/d bolus d1 to d5 every 3 weeks<br>Same + α-IFN 3 MU/d d-2 to d5 |
| Roma<br>Cassano et al, 1996 | 148 | 5FU 370 mg/m$^2$/d bolus d1 to d5, + l-folinic acid 80 mg/m$^2$/d bolus d1 to d5 every 4 weeks<br>Same + α-IFN 3 MU 3 times a week |
| Hungary<br>Pajkos et al, 1997 | 73 | 5FU 425 mg/m$^2$/d bolus d1 to d5 LV 20 mg/m$^2$/d d1 to d5 every 4 weeks<br>Same + α-IFN 3 MU three times a week |
| Argentina<br>Pensel et al, 1993 | 55 | 5FU 600 mg/m$^2$/d bolus d1 to d5 + LV 500 mg/m$^2$/d bolus d1 to d5 every 3 weeks<br>Same + α-IFN 5 MU/d, d1 to d5 every 3 weeks |
| **Comparison** | **Patients** | **Treatment arms** |
| **5FU + LV vs. 5FU + LV + α-IFN, with 5FU continuous infusion** | | |
| MRC<br>Seymour et al, 1996 | 260 | 5FU 800 mg/m$^2$/d, (bolus + continuous infusion) d1 and d2, + LV 200 mg/m$^2$/d bolus d1 and d2 every 2 weeks<br>Same + α-IFN 6 MU every other day d1 to d12 |
| AIO<br>Köhne et al, 1998 | 99 | 5FU = 2 600 mg/m$^2$/d IVC + LV = 500 mg/m$^2$/d bolus, every week<br>same + IFN = 3 MIU/d, 3d/w |

*© 2001 Cancer Research Campaign*

1998), trials were further stratified according to the duration of 5FU infusion. Bolus 5FU were administered in 5 comparisons (Hill et al, 1995; Dufour et al, 1996; Greco et al, 1996; Piga et al, 1996; Palmeri et al, 1998) and continuous infusion 5FU in one comparison (Hill et al, 1995b).

The comparison of 5FU + LV versus 5FU + LV + α-IFN was addressed in 6 trials, the Gruppo Oncologico dell'Italia Meridionale (GOIM) trial (Colucci et al, 1999), the Roma trial (Cassano et al, 1996), the trial from Hungary (Pajkos et al, 1997), the trial from Argentina (Pensel et al, 1993), the Medical Research Council (MRC) trial (Seymour et al, 1996), and the AIO trial (Köhne et al, 1998). The AIO trial (Köhne et al, 1998) and the trial from Hungary (Pajkos et al, 1997) were multiple-arm trials. Two trials (MRC (Seymour et al, 1996), AIO (Köhne et al, 1998)) used a continuous infusion 5FU. Trials were stratified according to 5FU schedule of administration (5FU bolus and 5FU continuous infusion), and in terms of modulation of 5FU by leucovorin.

### Meta-analysis of 5FU + LV vs. 5FU + α-IFN (Table 2)

The comparison of 5FU + LV versus 5FU + α-IFN was addressed in 7 trials, the Corfu-A trial (Corfu-A Study Group, 1995), the GOIRC trial (Di Costanzo et al, 1995), the Yale trial (Marsh et al,), the trial from Turkey (Aykan et al, 1996), the ECOG/CALGB trial (O'Dwyer et al, 1996), the AIO trial (Köhne et al, 1998), the trial Hungary (Pajkos et al, 1997). Three of these trials (O'Dwyer et al, 1996; Pajkos et al, 1997; Köhne et al, 1998) were multiple-arms trials.

In 4 trials same 5FU schedules were used in the 5FU/LV and in the 5FU+IFN arms: 5FU bolus in the GOIRC (Di Costanzo et al, 1995), the Hungary (Pajkos et al, 1997), and the Turkey (Aykan et al, 1996) trials, and 5FU continuous infusion in the AIO (Köhne et al, 1998). In the 3 remaining trials (Corfu-A Study Group, 1995); (Marsh et al) (O'Dwyer et al, 1996) 5FU consisted of bolus injection in the 5FU/LV arm, and of continuous infusion in the 5FU+IFN arm. Trials were therefore stratified according to 5FU administration, i.e. same 5FU schedules in both arms (Di Costanzo et al, 1995; Aykan et al, 1996, Pajkos et al, 1997; Kohne et al, 1998) or 5FU bolus vs. 5FU continuous infusion (Corfu-A Study Group, 1995; (Marsh et al,) (O'Dwyer et al, 1996).

### Protocol for the meta-analysis

In March 1997, all principal investigators received a protocol for the meta-analyses, and were asked to provide individual patient data. Information requested for every randomised patient was date of randomisation, tumour measurability (i.e. measurable or non-measurable tumours), treatment assigned by randomisation, age, gender, performance status according to the ECOG scale, primary tumour site (colon or rectum), prior adjuvant chemotherapy, prior chemotherapy for metastatic disease, site of metastases, overall response status with the first assigned treatment, date of response or progression with the first allocated treatment, cross-over to another treatment arm, date of death or last visit, survival status, and cause of death if applicable. Data on toxicity were not collected.

### Data collection

All individual patient data were received by April 1999. Data were extensively checked and discussed with all collaborators present at a plenary meeting of the *Meta-Analysis Group In Cancer* held in Atlanta, GA, in May 1999.

### Tumour response and survival

Complete response (CR) and partial response (PR) criteria adopted in individual trials followed the World Health Organization recommendations (Miller et al, 1981) and were similar in all trials. Patients experiencing minimal response, stable disease or progressive disease were considered to have no response for the purpose of the meta-analyses. In the MRC trial (Seymour et al, 1996) and in the trial from Hungary (Pajkos et al, 1997) chemotherapy was stopped after 6 months in the absence of tumour progression. In all

**Table 2** Randomised clinical trials comparing 5FU + LV to 5FU + α-IFN in advanced colorectal cancer

| Comparison | Patients | Treatment arms |
|---|---|---|
| **5FU + LV vs. 5FU + α-IFN, with the same dose of 5FU in both arms** | | |
| GOIRC<br>Di Costanzo et al, 1995 | 238 | 5FU 600 mg/m$^2$ bolus, + l-folinic acid 250 mg/m$^2$ bolus, + HU 3 g once a week for 6 weeks followed by a 2 week-break<br>Same without l-folinic acid + l-folinic acid + α-IFN 3 MU three times a week |
| AIO<br>Köhne et al, 1998 | 187 | 5FU 2 600 mg/m$^2$ continuous infusion + LV 500 mg/m$^2$ bolus, once a week for 6 weeks followed by a 2 week-break<br>Same without LV + α-IFN 3 MU three times a week |
| Turkey<br>Aykan et al, 1996 | 46 | 5FU 500 mg/m$^2$/d bolus d1 to d5 + l-folinic acid 100 mg/m$^2$, then weekly, every 4 weeks<br>Same without l-folinic acid + IFN 5 MU three times a week |
| **Comparison** | **Patients** | **Treatment arms** |
| **5FU + LV vs. 5FU + α-IFN, with a higher dose of 5FU in the 5FU + α-IFN arm** | | |
| Corfu-A<br>Corfu-A Study Group, 1995 | 496 | 5FU 370 mg/m$^2$/d bolus, + LV 200 mg/m$^2$/d d1 to d5 every 4 weeks<br>5FU 750 mg/m$^2$/d continuous infusion d1 to d5, then weekly on bolus + α-IFN 9 MU three times a week |
| ECOG/CALGB<br>O'Dwyer et al, 1996 | 443 | 5FU 600 mg/m$^2$/d bolus + LV 600 mg/m$^2$ bolus once a week<br>5FU 750 mg/m$^2$/d continuous infusion d1 to d5, then weekly on bolus + α-IFN 9 MU three times a week |
| Hungary<br>Pajkos et al, 1997 | 69 | 5FU 425 mg/m$^2$/d bolus d1 to d5 LV 20 mg/m$^2$/d d1 to d5 every 4 weeks<br>5FU 750 mg/m$^2$/d bolus d1 to d5 every 4 weeks + IFN 3 MU three times a week |
| Yale<br>Marsh et al | 9 | 5FU 425 mg/m$^2$/d bolus d1 to d5, + LV 20 mg/m$^2$/d d1 to d5 every 4 weeks<br>5FU 750 mg/m2/d continuous infusion d1 to d5, then weekly on bolus + α-IFN 9 MU three times a week |

other trials treatment was maintained until disease progression or severe toxicity. Duration of survival was calculated from the date of randomisation to the date of death, whatever its cause.

### Statistical methods

The statistical methods for meta-analyses based on individual patient data have been described in detail in previous publications (ACCMP, 1992; ACCMP, 1994; MAGIC, 1996; MAGIC, 1998a; MAGIC, 1998b). All analyses were based on an intention to treat basis, without any patient exclusion. Tumour responses were compared through relative risks (RR) in individual trials and overall (MAGIC, 1998b). Prognostic factors for response were identified through a logistic regression model (Cox, 1970). Survival times were compared through hazard ratios (HR) in individual trials and overall (Peto et al, 1977). Prognostic factors for survival were identified through a proportional hazards regression model (Cox, 1972). All P values were two-sided.

### RESULTS

### Patient characteristics

A total of 3254 were included in the analyses. The main patient characteristics are listed in Table 3 and 4. As could be expected in large series of patients, there was no imbalance between the experimental and the control groups for either of the comparisons of interest. 84% of patients had died at the time of analysis.

### Meta-analysis of 5FU ± LV vs. 5FU ± LV + α-IFN

1766 patients were included in this meta-analysis. The MRC trial (Seymour et al, 1996), the trial from Argentina (Pensel, 1993), and the trial from Ancona (Piga et al, 1996) allowed the inclusion of patients with non-measurable disease. After exclusion of these patients, 1683 patients were eligible for tumour response assessment. Relative risks for individual trials and overall are presented in Figure 1. Tumour response rates were 18% (70/387) for patients allocated to 5FU bolus alone and 21% (80/376) for patients allocated to 5FU bolus + α-IFN (RR = 0.86; 95%CI = 0.65–1.15). In the only trial using 5FU alone continuous infusion (Hill et al, 1995b), tumour response rate was 34% (27/80) for 5FU alone and 22% (18/80) for 5FU + α-IFN (RR = 1.50; 95% CI = 0.89–2.5).

Tumour response rates were 26% (59/227) for patients allocated to 5FU bolus + LV vs. 25% (59/233) for patients allocated to 5FU bolus + LV + α-IFN (RR = 0.99; 95% CI = 0.72–1.35), and 34% (52/151) for patients allocated to 5FU C.I + LV vs. 30% for patients allocated to 5FU C.I. + LV + α-IFN (RR = 1.14; 95% CI = 0.81–1.59). The overall tumour response rates were 25% (208/845) for 5FU ± LV, and 24% (202/838) for 5FU ± LV + α-IFN (RR = 1.02; 95% CI = 0.87–1.2; *P* = 0.8), showing no advantage for α-IFN administration.

There was no statistically significant survival difference between 5FU and 5FU + α-IFN, nor between 5FU + LV and 5FU + LV + α-IFN (Figure 2). The overall survival hazard ratio for the meta-analysis of 5FU ± LV versus 5FU ± LV + α-IFN was 0.95 (95% CI = 0.86–1.05; *P* = 0.33), showing no advantage for α-IFN

**Table 3** Patient characteristics: 5FU+/−LV vs. 5FU+/−LV+IFN

| Trial | Accrual period | Trt. | No. of patients | Adjuvant chemo. (%) | Primary colon (%) | PS<2 (%) | Metastases (%) Liver only | Metastases (%) Lung only |
|---|---|---|---|---|---|---|---|---|
| RICRC | 1989–92 | 5FU | 124 | 0 | NA | 83 | 62 | 9 |
| Greco et al, 1996 | | 5FU+IFN | 121 | 0 | NA | 92 | 63 | 4 |
| Palermo | 1990–93 | 5FU | 88 | 0 | 100 | 95 | 62 | 3 |
| Palmeri et al, 1998 | | 5FU+IFN | 81 | 0 | 100 | 97 | 61 | 0 |
| Ancona | 1990–93 | 5FU | 72 | 3 | 75 | 97 | 44 | 6 |
| Piga et al, 1996 | | 5FU+IFN | 69 | 3 | 72 | 97 | 45 | 6 |
| RMH | 1990–92 | 5FU | 54 | 0 | 63 | 87 | 28 | 2 |
| Hill et al, 1995a | | 5FU+IFN | 52 | 0 | 71 | 77 | 19 | 13 |
| France | 1990–93 | 5FU | 50 | 0 | 73 | 100 | 52 | 6 |
| Dufour et al, 1996 | | 5FU+IFN | 56 | 0 | 73 | 100 | 48 | 11 |
| RMH PVI | 1992–94 | 5FU | 80 | 0 | 81 | 58 | 19 | 9 |
| Hill et al, 1995b | | 5FU+IFN | 80 | 0 | 70 | 60 | 19 | 9 |
| GOIM | 1991–94 | 5FU/LV | 101 | 0 | 56 | 88 | 41 | 7 |
| Colucci et al, 1999 | | 5FU/LV+IFN | 103 | 1 | 66 | 95 | 40 | 2 |
| Roma | 1990–96 | 5FU/LV | 73 | 0 | 67 | 79 | 17 | 3 |
| Cassano et al, 1996 | | 5FU/LV+IFN | 75 | 0 | 71 | 81 | 10 | 3 |
| Hungary | 1993–96 | 5FU/LV | 35 | 0 | 47 | 74 | 60 | 0 |
| Pajkos et al, 1997 | | 5FU/LV+IFN | 38 | 0 | 66 | 76 | 39 | 3 |
| Argentina | 1990–91 | 5FU/LV | 28 | 0 | 61 | 57 | 43 | 0 |
| Pensel et al, 1993 | | 5FU/LV+IFN | 27 | 0 | 59 | 59 | 41 | 7 |
| MRC | 1991–93 | 5FU/LV | 132 | 1 | 69 | 74 | 43 | 3 |
| Seymour et al, 1996 | | 5FU/LV+IFN | 128 | 1 | 67 | 76 | 36 | 5 |
| AIO | 1992–93 | 5FU/LV | 50 | 10 | 46 | 94 | 34 | 0 |
| Köhne et al, 1998 | | 5FU/LV+IFN | 49 | 6 | 51 | 96 | 44 | 4 |
| Total | 1989–96 | 5FU+/-LV | 887 | 1 | 70 | 98 | 43 | 5 |
| | | 5FU+/-LV+IFN | 879 | 1 | 71 | 98 | 40 | 5 |

NA = not available.

*© 2001 Cancer Research Campaign*

**Table 4** Patient characteristics: 5FU+LV vs. 5FU+IFN

| Trial | Accrual Period | Trt. | No.of Patients | Adjuvant Chemo. (%) | Primary colon (%) | PS<2 (%) | Metastases (%) Liver only | Metastases (%) Lung only |
|---|---|---|---|---|---|---|---|---|
| Corfu-A | 1989–91 | 5FU/LV | 250 | 0 | NA | 83 | 38 | 4 |
| Corfu-A Study Group, 1995 | | 5FU+IFN | 246 | 0 | NA | 83 | 37 | 4 |
| ECOG | 1990–95 | 5FU/LV | 224 | 12 | 68 | 92 | 37 | 11 |
| O'Dwyer et al, 1996 | | 5FU+IFN | 219 | 11 | 73 | 95 | 35 | 9 |
| AIO | 1992–95 | 5FU/LV | 93 | 12 | 50 | 96 | 44 | 4 |
| Köhne et al, 1998 | | 5FU+IFN | 94 | 11 | 61 | 92 | 39 | 7 |
| GOIRC | 1992–94 | 5FU/LV | 119 | 0 | 64 | 98 | 56 | 4 |
| Di Costanzo et al, 1995 | | 5FU+IFN | 119 | 0 | 59 | 97 | 59 | 5 |
| Hungary | 1993–96 | 5FU/LV | 35 | 0 | 47 | 74 | 60 | 0 |
| Pajkos et al, 1998 | | 5FU+IFN | 34 | 0 | 53 | 79 | 50 | 3 |
| Turkey | 1992–94 | 5FU/LV | 19 | 15 | 50 | 72 | 21 | 0 |
| Aykan et al, 1996 | | 5FU+IFN | 27 | 15 | 21 | 62 | 37 | 11 |
| Yale | 1990–91 | 5FU/LV | 4 | 0 | 75 | 100 | 25 | 25 |
| Marsh et al | | 5FU+IFN | 5 | 0 | 100 | 80 | 60 | 0 |
| Total | 1989–96 | 5FU/LV | 744 | 6 | 61 | 89 | 42 | 6 |
| | | 5FU+IFN | 744 | 5 | 64 | 89 | 41 | 6 |

NA = not available.

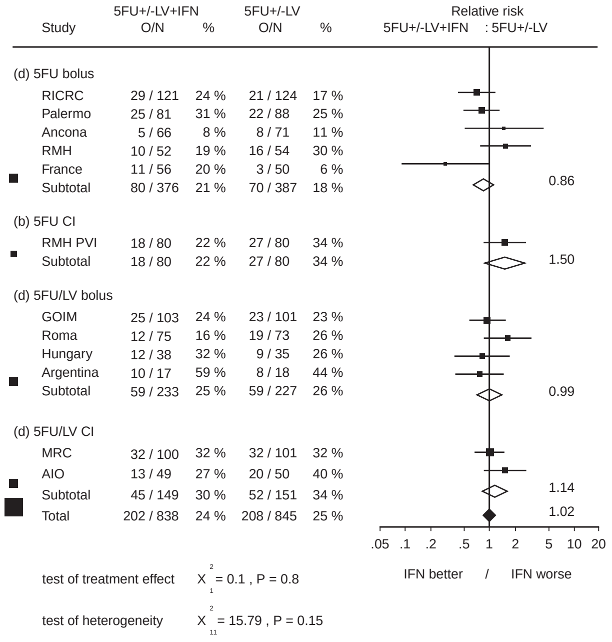

**Figure 1** Tumour response relative risks in individual trials and overall for the meta-analysis 5FU ± LV vs. 5FU ± LV + α-IFN

administration. Median survivals were 11.4 months for patients treated without α-IFN, and 11.5 months for patients treated with α-IFN.

### Meta-analysis of 5FU + LV vs. 5FU + α-IFN

1488 patients were included in this meta-analysis.

The ECOG/CALGB trial (O'Dwyer et al 1996) allowed the inclusion of patients with non-measurable disease. After exclusion of these patients, 1305 patients were eligible for tumour response assessment.

Tumour response rates were 23% (152/655) for patients allocated to 5FU + LV vs. 18% (115/650) for patients allocated to 5FU + α-IFN. The overall tumour response RR was 1.26 (95% CI = 1.01–1.59; $P = 0.042$), showing a statistically significant advantage for 5FU + LV over 5FU + α-IFN (Figure 3). However, the heterogeneity between trials in this meta-analysis was rather important ($P$ value for heterogeneity, $P = 0.001$), mostly between trials using the same 5FU schedules in both treatment arms ($P$ value for heterogeneity, $P = 0.003$).

Analyses stratified by type of 5FU administration showed that the advantage of 5FU + LV over 5FU + α-IFN was limited to the group of trials using the same 5FU schedules in both treatment arms (RR = 1.80; 95% CI = 1.29–2.51; $P = 0.0005$).

Survival analysis showed a small trend in favour of 5FU + LV over 5FU + α-IFN, but this advantage was not statistically significant (overall HR = 1.11; 95% CI = 0.99–1.24; $P = 0.066$) (Figure 4). Median survivals were 11.7 months for patients allocated to 5FU + LV and 11.3 months for patients allocated to 5FU + α-IFN. The survival difference reached statistical significance in the group of trials using the same 5FU schedules in both treatment arms (HR = 1.29; 95% CI 1.07–1.57; $P = 0.008$). There was some heterogeneity in this group of trials, but which did not reach a statistically significant level ($P = 0.67$).

### Prognostic factor analyses

Individual patient data used for the two meta-analyses were combined to identify prognostic factors for response and survival (3254 patients). Sex, age, performance status (PS), primary tumour site, previous adjuvant chemotherapy, metastatic site, and allocated treatment (no α-IFN vs. α-IFN) were considered in these analyses. In a logistic regression model, metastases confined to the liver ($P < 10^{-4}$), and primary rectal tumours ($P = 0.042$) were the independent favourable prognostic factors for tumour response. Tumour response rates were 26% for patients with metastases confined to the liver versus 20% for the others. Patients with rectal cancer had a 26% tumour response rate, vs. 22% with colon tumour.

© 2001 Cancer Research Campaign 

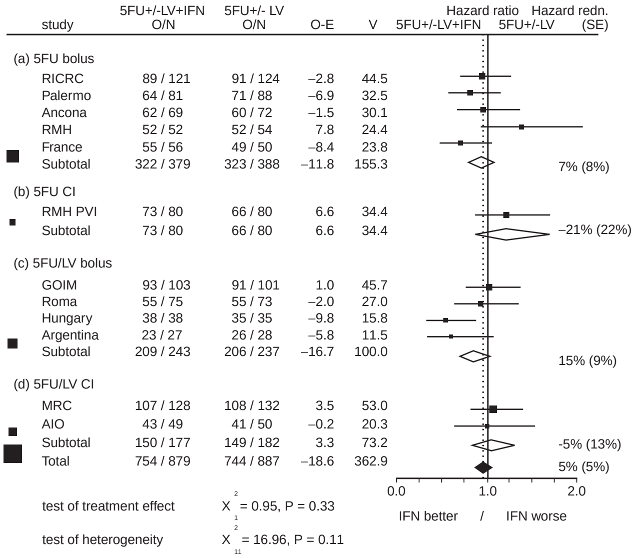

**Figure 2** Survival hazard ratios in individual trials and overall for the meta-analysis 5FU ± LV vs. 5FU ± LV + α-IFN

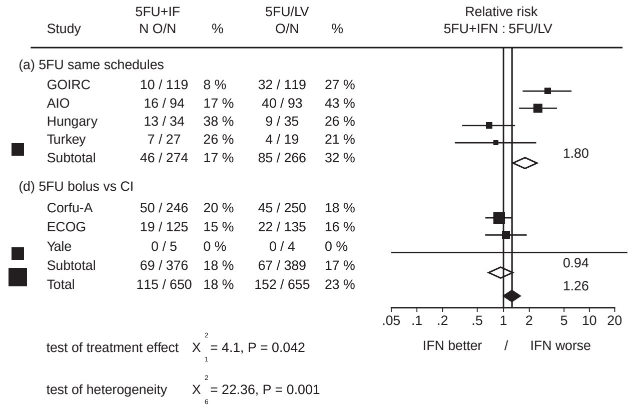

**Figure 3** Tumour response relative risks in individual trials and overall for the meta-analysis 5FU + LV vs. 5FU + α-IFN

In a Cox regression model, good PS ($P < 10^{-4}$), metastases confined to the liver or confined to the lung ($P$ = 0.0002 and $P$ = 0.004 respectively), and primary tumour in the rectum ($P$ = 0.003) were the independent favourable prognostic factors for survival. One-year survival was 59% for patients with PS 0, 47% for patients with PS 1, and 30% for patients with PS 2 or worse; 54% for patients with metastases confined to the liver, 46% for the others; 61% for patients with metastases confined to the lung, 49% for the others; 56% for patients with primary rectal cancer, 47% for the others.

### DISCUSSION

Pre-clinical studies indicate that α-IFN may increase the cytotoxicity of 5FU in a variety of tumour cell lines (Elias and Crissman, 1988; Wadler et al, 1990). Several mechanisms of interaction between 5FU and interferon have been demonstrated. In vitro data published by Elias and Crissman (Elias and Crissman, 1988) suggest that the enzyme thymidylate synthase might be a target for this interaction. Moreover, the presence of thymidine in the culture

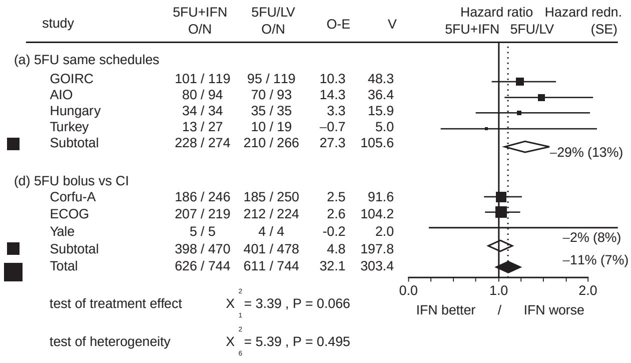

**Figure 4** Survival hazard ratios in individual trials and overall for the meta-analysis 5FU + LV vs. 5FU + α-IFN

medium tends to block the synergic effect (Neefe and John, 1991). Interferon may also modify the plasma pharmacokinetics of 5FU (Lindley et al, 1990; Danhouser et al, 1991). Finally, 5FU may influence the immunomodulatory actions of interferon (Neefe and John, 1991). However, despite more than 3000 patients included in randomized trials, the clinical impact of combining α-IFN to 5FU remained debatable.

The 2 meta-analyses presented here address the efficacy of α-IFN combined with 5FU in advanced colorectal cancer. Tumour response rate and survival were the two main end points. Toxicity was not studied, since at the time of beginning these meta-analyses individual trials had already demonstrated that the addition of α-IFN to a 5FU regimen led to an increased risk of neutropenia, mucositis, and neurotoxicity, and was associated with flu-like syndromes. α-IFN also produced a significant impairment of quality of life in the MRC trial (Seymour et al, 1996).

The meta-analysis of trials comparing 5FU ± LV to a similar 5FU regimen plus α-IFN failed to show any difference between control and experimental arms in terms of tumour response or survival. The tumour response rate with 5FU bolus alone reported in the group of trials comparing 5FU to 5FU + α-IFN was rather high (19%), compared to tumour responses reported for patients receiving 5FU bolus in the 4 meta-analyses previously performed by our group, which varied between 11% and 14% (ACCMP, 1992, 1994; MAGIC, 1996, 1998a). This may reflect a selection of patients with favourable prognostic characteristics in trials included in the present meta-analysis, but does not invalidate our finding of no difference between 5FU alone and 5FU + α-IFN. It should also be noted that the doses of 5FU delivered in the 5FU alone arms were generally high compared with the 5FU doses reported in our previous meta-analyses.

In contrast, the meta-analysis of trials comparing 5FU + LV to 5FU + α-IFN showed higher response rates and a trend towards longer survival in favour of 5FU + LV. In this set of trials, the overall tumour response rate and the median survival of patients receiving 5FU + LV (23% and 13 months, respectively) were remarkably similar to those reported previously in the meta-analysis of trials comparing 5FU to 5FU + LV (ACCMP, 1992), (23% and 11.5 months, respectively). Thus, the advantage of 5FU + LV over 5FU + α-IFN observed in the present meta-analysis does not seem to be due to some selection bias that might have favoured patients allocated to the 5FU + LV arm.

In this meta-analysis, the stratification of trials by type of 5FU administration (Figures 3 and 4) showed a statistically significant

 *© 2001 Cancer Research Campaign*

advantage of 5FU/LV over 5FU+IFN in the group of trials using the same 5FU schedules in both arms. By contrast, there was no difference between the two treatment arms when 5FU was administered by bolus in the 5FU/LV arm and by continuous infusion in the 5FU + IFN. This could be linked to the tumour response and survival advantage of 5FU continuous infusion over 5FU bolus demonstrated in one of our previous meta-analyses (MAGIC, 1998a).

5FU dose intensity is not a valid parameter when comparing bolus versus infusion or mixed regimens. Consequently, no attempt was made to stratify trials according to 5FU dose intensity.

The prognostic factor analysis confirms well-established results, such as the key role of performance status for survival. Other findings are less classical, such as the role of primary and metastatic tumour sites, and are currently under investigation by our group, on the basis of 7000 individual patient data with advanced colorectal cancer. In the adjuvant setting, a trial conducted by the *National Surgical Adjuvant Breast and Bowel Project* (NSABP-C05) also failed to show any advantage for 5FU + LV + α-IFN over 5FU + LV in patients with stage II-III colon cancer (Wolmark et al, 1998). On-going studies are currently addressing the interest of other types of interferon, such as α-2c IFN and β-IFN (Villar Grimalt et al, 1999). However, new agents, such as CPT-11 (irinotecan) (Douillard et al, 2000; Saltz et al, 2000) or oxaliplatin (de Gramont et al, 2000) have demonstrated clinical benefits in advanced colorectal cancer, and are therefore more plausible candidates for the adjuvant setting.

We conclude that α-IFN does not increase the efficacy of 5FU in advanced colorectal cancer, and should not be offered in routine clinical practice.

### ACKNOWLEDGMENTS

This study was supported in part by the European Association for Research in Oncology (A.E.R.O.), France, and by Roche Laboratories. The authors thank Youri Piedbois for data management and statistical support.

### REFERENCES

Advanced Colorectal Cancer Meta-analysis Project (1992) Modulation of 5-fluorouracil by leucovorin in patients with advanced colorectal cancer: evidence in terms of response rate. *J Clin Oncol* **10**: 896–903

Advanced Colorectal Cancer Meta-analysis Project (1994) Meta-analysis of randomized trials testing the biochemical modulation of 5-fluorouracil by methotrexate in metastatic colorectal cancer. *J Clin Oncol* **12**: 960–969

Aykan NF, Uskent N, Yaylaci M, et al (1996) 5-fluorouracil plus interferon alpha-2b versus 5-fluorouracil plus folinic acid in advanced colorectal cancer; same efficacy but different toxicity. *Ann Oncol* **7** (suppl 5): 42 (abstract 195)

Cassano A, Pozzo C, Corsi DC, et al (1996) The role of interferon alpha-2b in the treatment with folinic acid and 5-fluorouracil of advanced colorectal cancer: a randomized study. 9th NCI-EORTC symposium on new drugs in cancer therapy; March 12–15, 1996, Amsterdam. *Ann Oncol* **7** (Suppl 1): 69 (abstract 234)

Colucci G, Maiello E, Gebbia V et al (1999) 5-Fluorouracil and Levofolinic acid with or without recombinant interferon-2b in patients with advanced colorectal carcinoma. A randomized multicenter study with stratification for tumor burden and liver involvement by the Southern Italy Oncology Group. *Cancer* **85**: 535–545

Corfu-A Study Group (1995) Phase III randomized study of two fluorouracil combinations with either interferon alpha-2a or leucovorin for advanced colorectal cancer. *J Clin Oncol* **13**: 921–928

Cox DR (1970) The analysis of binary data. London, United Kingdom, Methuen.

Cox DR (1972) Regression models and life tables (with discussion). *JR Stat Soc B* **34**: 187–220

Danhauser L, Gilchrist T, Friemann J et al. (1991) Effect of recombinant interferon-alpha-2b on the plasma pharmacokinetics of fluorouracil in patients with advanced cancer. *Proc Am Assoc Cancer Res* **32**: 1052 (abstr)

de Gramont A, Figer A, Seymour M, Homerin M, Hmissi A, Cassidy J, Boni C, Cortes-Funes H, Cervantes A, Freyer G, Papamichael D, Le Bail N, Louvet C, Hendler D, de Braud F, Wilson C, Morvan F and Bonetti A (2000) Leucovorin and fluorouracil with or without oxaliplatin as first-line treatment in advanced colorectal cancer. *J Clin Oncol* **18**: 2938–2947

Di Costanzo F, El-Taani H, Marzola M, et al (1995) Hydroxyurea (HU), high dose folinic acid (1-FA) and 5FU vs HU, 5FU and interferon-alfa-2b (IFN) in advanced colorectal cancer (ACRC): a randomized trial of the Italian Oncology Group for Clinical Research (GOIRC). *Proc Am Soc Clin Oncol* **14**: 208 (abstract 508)

Douillard JY, Cunningham D, Roth AD, Navarro M, James RD, Karasek P, Jandik P, Iveson T, Carmichael J, Alakl M, Gruia G, Awad L and Rougier P (2000). Irinotecan combined with fluorouracil compared with fluorouracil alone as first-line treatment for metastatic colorectal cancer: a multicentre randomised trial. *Lancet* **355**: 1041–1047

Dufour P, Husseini F, Dreyfus B et al (1996) 5-Fluorouracil *versus* 5-Fluorouracil plus alpha-interferon as treatment of metastatic colorectal carcinoma. A randomized study. *Ann Oncol* **7**: 575–579.

Elias L and Crissman HA (1988) Interferon effects upon the adenocarcinoma 38 and HL-60 cell lines: antiproliferative responses and synergistic interactions with halogenated pyrimidine antimetabolites *Cancer Res* **48**: 4868–4873

Greco FA, Figlin R, York M et al (1996) Phase III randomized study to compare interferon alpha-2a in combination with fluorouracil versus fluorouracil alone in patients with advanced colorectal cancer. *J Clin Oncol* **14**: 2674–2681

Hill M, Norman A, Cunningham D et al (1995a): Royal Marsden phase III trial of fluorouracil with or without interferon Alfa-2b in advanced colorectal cancer. *J Clin Oncol* **13**: 1297–1302

Hill M, Norman A, Cunningham D et al (1995b) Impact of protracted venous infusion fluorouracil with or without interferon alpha-2b on tumor response, survival, and quality of life in advanced colorectal cancer. *J Clin Oncol* **13**: 2317–2323

Köhne CH, Schöffski P, Wilke H et al (1998) Effective biomodulation by leucovorin or high-dose infusion fluorouracil given has a weekly 24-hour infusion: results of a randomized trial in patients with advanced colorectal cancer. *J Clin Oncol* **16**: 418–426

Kosmidis PA, Tsavaris N, Skarlos D et al (1996) Fluorouracil and leucovorin with or without interferon alfa-2b in advanced colorectal cancer: analysis of a prospective randomized phase III trial. Hellenic Cooperative Oncology Group. *J Clin Oncol* **14**: 2682–2687

Kreuser ED, Streit M, Kuchler T et al (1995) A multicenter randomized trial with the assessment of quality of life in patients with metastatic colorectal carcinoma given either folinic acid or interferon α-2b as a modulator of 5-fluorouracil. *Prc Am Soc Clin Oncol* **14**: 202 (abstract 485)

Lindley C, Bernard B, Gavlan M et al (1990) Interferon-alpha increases 5-fluorouracil levels 16-fold within 1 hour: results of a phase I study. *J Interferon Res* **10**: 9–15

Marsh JC and Rosenberg AH, YALE-HIC-5698, NCI-V91-0053. Phase III comparison of 5FU/low-dose CF vs 5FU/IFN-A in patients with metastatic or recurrent colorectal adenocarcinoma. PDQ Notice

Meta-Analysis Group In Cancer (1996) Reappraisal of hepatic arterial infusion in the treatment of non resectable liver metastases from colorectal cancer. *J Natl Cancer Inst* **88**: 252–258

Meta-Analysis Group In Cancer (1998a) Efficacy of intravenous continuous infusion of fluorouracil compared with bolus administration in advanced colorectal cancer. *J Clin Oncol* **16**: 301–308

Meta-Analysis Group In Cancer (1998b) Toxicity of fluorouracil in patients with advanced colorectal cancer: effect of administration schedule and prognostic factors. *J Clin Oncol* **16**: 3537–3541

Miller AB, Hoogstraten B, Staquet M and Winkler A (1981) Reporting results of cancer treatment. *Cancer* **47**: 207–214

Neefe JR and John W (1991) Mechanisms of interaction of interferon and 5-fluorouracil in solid tumors. *Semin Oncol* **18** (suppl 7): 77–82

O'Dwyer PJ, Ryan LM, Valone FH et al (1996) Phase III of biochemical modulation of 5-fluorouracil by IV or oral leucovorin or by interferon in advanced colorectal cancer: an ECOG/CALGB phase III trial. *Proc Am Soc Clin Oncol* **15**: 207 (abstract 469).

Pajkos G, Izso J, Kristo K et al (1997) Biochemical modulation of 5-fluorouracil (FU) by leucovorin (LV) and/or interferon alpha-2a (IFN) in metastatic colorectal cancer (MCC). Anti-Cancer Treatment, Seventh International Congress 1997, Paris, February 6–9, 1997: 194 (abstract 275)

Palmeri S, Danova M, Bernardo G et al (1998) 5-Fluorouracil plus interferon alpha-2a compared to 5-Fluorouracil alone in the treatment of advanced colon carcinoma: a multicentric randomized study. *J Cancer Res Clin Oncol* **124**: 191–198

Pazdur R, Ajani JA, Patt YT et al (1990) Phase II study of fluorouracil and recombinant interferon alpha-2a in previously untreated advanced colorectal carcinoma. *J Clin Oncol* **10**: 2027–2031

Pensel R (1993). Advanced colon cancer (ACC): a randomized trial of fluorouracil (5FU)+folinic acid (FA) and 5FU+FA+Interferon alpha-2b (IFN). *Proc Am Soc Clin Oncol* **12**: 203 (abstract 602)

Peto R, Pike MC, Armitage P et al (1977) Design and analysis of randomized clinical trials requiring prolonged observation of each patient (II. Analysis and examples). *Br J Cancer* **35**:1–39

Piedbois P, Gimonet JF, Feuilhade F et al (1991) 5FU, folinic acid and alpha-2a-interferon combination in advanced gastrointestinal cancer. *Proceedings of the American Society of Clinical Oncology* **10**: 430

Piga A, Cascinu S, Latini L et al (1996) A phase II randomised trial of 5-fluorouracil with or without interferon alpha-2a in advanced colorectal cancer. *B J Cancer* **74**: 971–974

Raderer M and Scheithauer W (1995) Treatment of advanced colorectal cancer with 5-fluorouracil and interferon-alpha: an overview of clinical trials. *Eur J Cancer* **31A**: 1002–1008

Recchia F, Nuzzo A, Lalli A et al (1996) Randomized trial of 5-fluorouracil and high dose folinic acid with or without alpha-2b interferon in advanced colorectal cancer. *Am J Clin Oncol* **19**: 301–304

Saltz LB, Cox JV, Blanke C, Rosen LS, Fehrenbacher L, Moore MJ, Maroun JA, Ackland SP, Locker PK, Pirotta N, Elfring GL and Miller LL (2000) Irinotecan plus fluorouracil and leucovorin for metastatic colorectal cancer. Irinotecan Study Group. *N Engl J Med* **343**: 905–914

Seymour MT, Slevin ML, Kerr DJ et al (1996) Randomized trial assessing the addition of interferon alpha-2a to fluorouracil and leucovorin in advanced colorectal cancer. *J Clin Oncol* **14**: 2280–2288

Villar-Grimalt A, Candel MT, Massuti B et al (1999) A randomized phase II trial of 5-fluorouracil, with or without human interferon-beta, for advanced colorectal cancer. *Br J Cancer* **80**(5–6): 786–791

Wadler S, Schwartz EL, Goldman M et al (1989) Fluorouracil and recombinant alpha-2a-interferon: an active regimen against advanced colorectal carcinoma. *J Clin Oncol* **12**: 1769–1775

Wadler S, Wersto R, Weinberg V et al (1990) Interaction of fluorouracil and interferon in human colon cancer cell lines: cytotoxic and cytokinetic effects. *Cancer Res* **50**: 2653–2665

Weh HJ, Platz D, Braumann D et al (1992) Treatment of metastatic colorectal carcinoma with a combination of fluorouracil and recombinant interferon alpha-2b: preliminary-data of a phase II study. *Semin Oncol Suppl* **2**: 180–184

Wolmark N, Bryant J, Smith R et al (1998) Adjuvant 5-fluorouracil and leucovorin with or without interferon alfa-2a in colon carcinoma: National Surgical Adjuvant Breast and Bowel Project protocol C-05. *J Natl Cancer Inst* **90**(23): 1810–1816

*© 2001 Cancer Research Campaign*

### AUTHORS' NAMES & ADDRESSES

Pierre Thirion, MD
Department of Radiotherapy
Saint Luke's Hospital
Highfield Road
Rathgar
DUBLIN 6
IRELAND

Pascal Piedbois, MD, PhD
Department of Medical Oncology
Hôpital Henri Mondor
Assistance Publique – Hôpitaux de Paris
51 avenue du Maréchal de Lattre de Tassigny
F-94010 CRÉTEIL CEDEX
FRANCE

Marc Buyse, ScD
International Drug Development Institute
430 avenue Louise, B14
B-1050 BRUSSELS
BELGIUM

Peter J. O'Dwyer, MD
Fox Chase Cancer Center
Section of Urology
1500 E Medical Center Dr.
Ann Arbor, MI 48109–0330
USA

David Cunningham, MD
Cancer Research Campaign, Section of Medicine
The Royal Marsden Hospital
Downs Road
SUTTON
Surrey
SM2 5PT
UNITED KINGDOM

Anthony Man, MD
Department of Clinical Pharmacology and Clinical Research
Novartis Pharma
CH-4002 BASEL
SWITZERLAND

Frank A. Greco, MD
Sarah Cannon Cancer Center
250 25th Avenue North
Suite 412
NASHVILLE, TN 37203–1632
USA

Giuseppe Colucci, MD
Department of Medicine
Oncology Institute
Via Amendola, 209
I-70126 BARI
ITALY

Claus-Henning Köhne, MD
Abt. für Hämatologie, Onkologie and Tumorimmunologie
Virchow-Klinikum
Robert-Rössle-Klinik
Linderberger Weg 80
D-13122 BERLIN
GERMANY

Francesco Di Costanzo, MD
Servicio Oncoligico
Azienda Ospedaliera "S. Maria"
Via T. di Joannuccio
I-05100 TERNI
ITALY

Andrea Piga, MD
Medical Oncology
University of Ancona
Ospedale Torrette
I-60020 ANCONA
ITALY

Sergio Palmeri, MD
Ass. Professor of Medical Oncology
Istituto di Clinica Medica, University of Palermo
Policlinico "P. Giaccone", p.zza delle Cliniche, 2
I-90127 PALERMO
ITALY

Patrick Dufour, MD
Département OncoHématologie
CHU Hautepierre
Avenue Molière
F-67098 STRASBOURG CEDEX
FRANCE

Alessandra Cassano, MD PhD
Department of Internal Medicine/Medical Oncology
Universita Catolica S. Cuore
Largo A. Gemelli 8
I-00168 ROMA
ITALY

Gábor Pajkos, M.D.
MI Central Hospital
2nd Dept. Internal Medicine-Oncology
BUDAPEST
1406 P.O.B. 23
HUNGARY

Raul Augusto Pensel, MD
Centro Oncologico "Dr Alejandro Catarineu"

© 2001 Cancer Research Campaign

Carlos Calvo 1777
1102 Buenos Aires
ARGENTINA

N. Faruk Aykan, MD
Istanbul University
Institute of Oncology
34390 ÇAPA, ISTANBUL
TURKEY

John Marsh, MD
Yale Comprehensive Cancer Center
P.O. Box 208032
333 Cedar Street
NEW HAVEN, CT 06520–8032
USA

Matthew T. Seymour
University of Leeds
Cookridge Hospital
Regional Centre for Cancer Treatment
LEEDS
LS16 6QB
UNITED KINGDOM

 *© 2001 Cancer Research Campaign*
